# Developing and evaluating clinical leadership interventions for frontline healthcare providers: a review of the literature

**DOI:** 10.1186/s12913-018-3561-4

**Published:** 2018-10-01

**Authors:** Solange Mianda, Anna Voce

**Affiliations:** Discipline of Public Health Medicine, Room 236, 2nd floor George Campbell Building, School of Nursing and Public Health, College of Health Sciences, University of KwaZulu-Natal, Durban, South Africa

**Keywords:** Clinical leadership development, Clinical leadership evaluation, Frontline healthcare providers, Bedside

## Abstract

**Background:**

The importance of clinical leadership in ensuring high quality patient care is emphasized in health systems worldwide. Of particular concern are the high costs to health systems related to clinical litigation settlements. To avoid further cost, healthcare systems particularly in High-Income Countries invest significantly in interventions to develop clinical leadership among frontline healthcare workers at the point of care. In Low-Income Countries however, clinical leadership development is not well established. This review of the literature was conducted towards identifying a model to inform clinical leadership development interventions among frontline healthcare providers, particularly for improved maternal and newborn care.

**Methods:**

A structural literature review method was used, articles published between 2004 and 2017 were identified from search engines (Google Scholar and EBSCOhost). Additionally, electronic databases (CINHAL, PubMed, Medline, Academic Search Complete, Health Source: Consumer, Health Source: Nursing/Academic, Science Direct and Ovid®), electronic journals, and reference lists of retrieved published articles were also searched.

**Results:**

Employing pre-selected criteria, 1675 citations were identified. After screening 50 potentially relevant full-text papers for eligibility, 24 papers were excluded because they did not report on developing and evaluating clinical leadership interventions for frontline healthcare providers, 2 papers did not have full text available. Twenty-four papers met the inclusion criteria for review. Interventions for clinical leadership development involved the development of clinical skills, leadership competencies, teamwork, the environment of care and patient care. Work-based learning with experiential teaching techniques is reported as the most effective, to ensure the clinical leadership development of frontline healthcare providers.

**Conclusions:**

All studies reviewed arose in High-Income settings, demonstrating the need for studies on frontline clinical leadership development in Low-and Middle-Income settings. Clinical leadership development is an on-going process and must target both novice and veteran frontline health care providers. The content of clinical leadership development interventions must encompass a holistic conceptualization of clinical leadership, and should use work-based learning, and team-based approaches, to improve clinical leadership competencies of frontline healthcare providers, and overall service delivery.

## Background

Clinical leadership by frontline healthcare providers is a critical part of bedside care [1]. Clinical leadership is recommended for the potential impact on clinical practice and on the clinical care environment, and contributes to safe and quality patient care, and to job satisfaction and retention of frontline healthcare providers [1–6]. Frontline healthcare providers are well placed to identify work inefficiencies, motivate other members of the care team to act on patient care, and lead change initiatives to correct problems that arise in the clinical setting. Frontline healthcare providers can also identify inefficiencies related to organizational structures and work flows, and to poor policies and procedures for the delivery of optimal patient care [2, 5, 7–10]. Conversely, poor frontline clinical leadership in the clinical setting has been associated with adverse events and clinical litigation settlements, prompting many healthcare systems, particularly in High-Income Countries (HICs), to invest significantly in interventions that support clinical leadership development [3, 11].

However, in Low- and Middle-Income Countries (LMICs), clinical leadership development is not well established. As an example, in South African maternity services, maternal and perinatal deaths have been associated with deficiencies in frontline clinical leadership [12–18]. Albeit the need for clinical leadership development interventions has been identified, there is little evidence to support the planning, implementation and evaluation of such interventions, particularly among frontline healthcare providers, in LMICs [13–18].

Towards identifying a model to inform clinical leadership development among frontline healthcare providers in LMIC, including maternity services in South Africa, a literature review was conducted. The purpose of the literature review was to synthesize published evidence on frontline clinical leadership development and its evaluation and included multiple frontline-care contexts. A database was constructed to extract important dimensions of the clinical leadership development interventions. Further, to synthesize the reported findings on the evaluation of the effectiveness of clinical leadership interventions, Kirkpatrick’s evaluation approach was used [19, 20]. Kirkpatrick’s approach to evaluation comprises four levels, presented as a sequence, and includes evaluating the:

1. Reaction: what participants think and feel about the intervention
2. Learning: the resulting increase in knowledge or skills, and changes in attitude
3. Behaviour: change in practice because of the intervention
4. Result: the final result that occurs as a result of the intervention (e.g. service delivery, or patient outcomes) [19, 20].

The findings of the literature review will contribute to the design and evaluation of interventions to improve clinical leadership at the bedside in LMICs generally, and in the maternity services of South Africa specifically.

## Methods

### Aim

The aim of the literature review was to describe the characteristics and the evaluation of clinical leadership development interventions targeting frontline healthcare providers.

### Design

A structured approach, the systematic quantitative literature review method [21], was used to search and identify the literature, and extract information on interventions for clinical leadership development.

### Search methods

The searches were conducted using Google Scholar and EBSCOhost search engines. Additionally, electronic databases including CINHAL, PubMed, Medline, Academic Search Complete, Health Sources: Nursing/Academic Edition, Science Direct and Ovid®), were searched using the following keywords: ‘clinical leadership’, ‘frontline leadership’, ‘nursing leadership’, ‘ward leadership’, ‘medical leadership’, ‘clinician leadership’ in combination with: ‘development’, ‘programme’, ‘interventions’, ‘evaluation’ and ‘training’. A manual search was conducted to trace sources in the reference list of retrieved published articles.

#### Eligibility criteria

##### Inclusion criteria

Papers meeting the following criteria were included for review: (1) original research published in peer-reviewed journals; (2) grey literature; (3) reporting the implementation or evaluation of interventions for clinical leadership development; (4) published in English between 2004 and 2017.

##### Exclusion criteria

Papers exploring the implementation and/or the evaluation of interventions or approaches for the development of health service or organizational leadership, or development of senior healthcare leaders were excluded.

### Assessment of publications

The database search generated 1600 records; grey literature (health services reports, research reports, theses, and dissertations) generated 75 records; of which 1558 were duplicate. On a review of abstracts 117 papers were excluded [related to developing or evaluating organizational or health services leadership]. On screening 50 potentially relevant full-text papers, 24 were excluded [did not report on developing or evaluating clinical leadership for frontline healthcare providers], and 2 [did not have full text available]. Twenty-four papers met the inclusion criteria and were captured in the database.

**Fig. 1:**
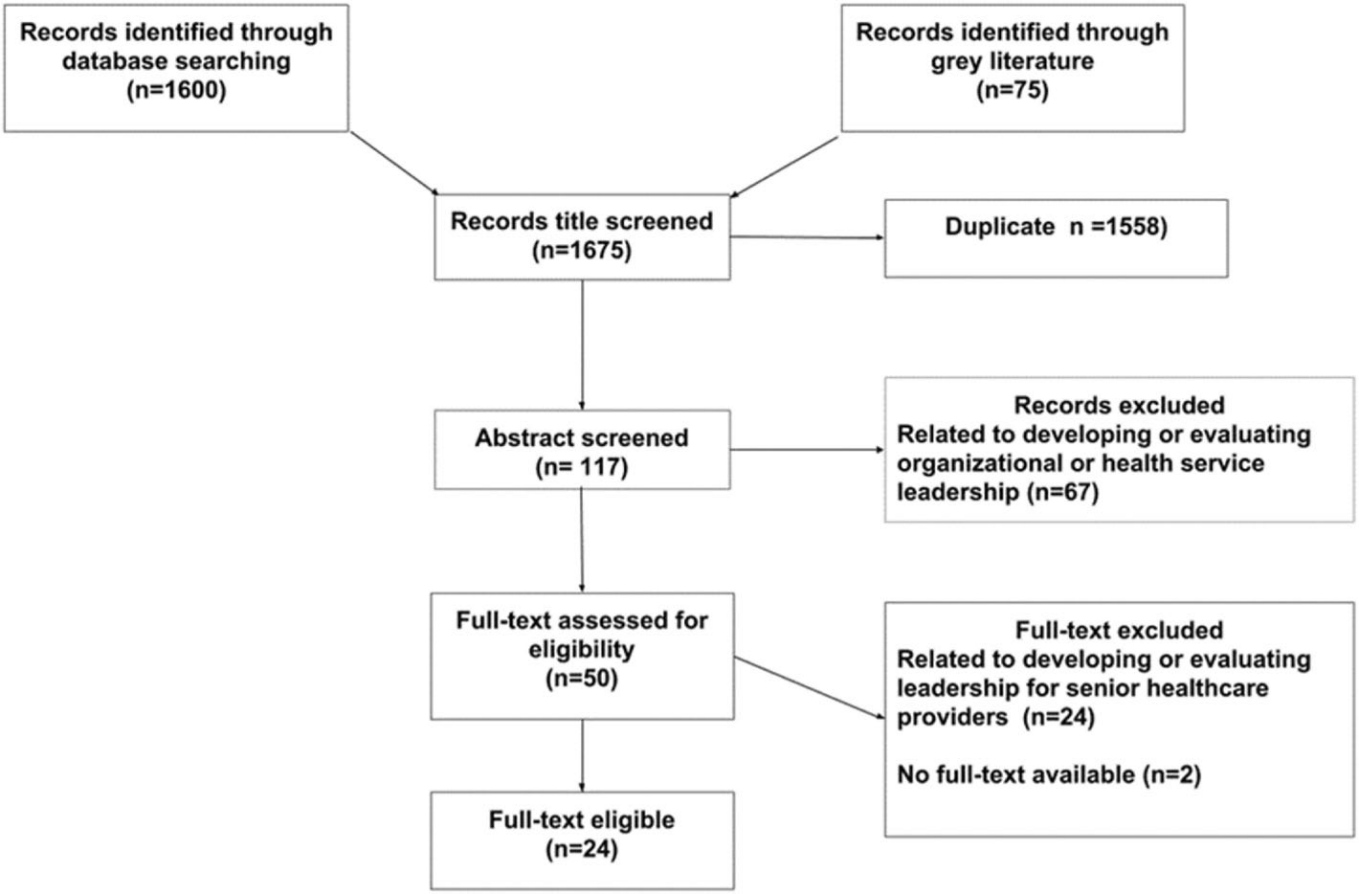
Search algorithm, indicating number of identified studies, included and excluded studies, and reasons for exclusion

Figure 1 presents the search algorithm indicating the number of identified studies, included and excluded studies, and reasons for exclusion.

The quality of the studies reviewed was appraised using the Standards for Reporting Implementation Studies (StaRI) [22]. Against the StaRI criteria, the studies reporting the interventions and the evaluation of interventions for clinical leadership development did not provide adequate descriptions of the interventions themselves, of the methods used in implementing the interventions, and of the evaluation of the interventions. However, they included sound descriptions of the aims and the target groups for which the interventions were designed. Two studies provided sufficient descriptions of the intervention, the implementation and the evaluation in order to produce transferable findings [23, 24]. Overall, the studies included in this review were of poor quality. However, the shortcomings identified did not detract from the purpose of the present literature review.

### Constructing the database

A database was constructed to summarise the studies identified for the review. The following information was captured in the database: the country where the intervention/evaluation was implemented, the aim of the intervention/evaluation, the target population for which the intervention was designed, the content areas of the intervention, the educational approach used, the educational techniques used, the time frame of the intervention, how the impact of intervention was measured, the outcomes and limitations of the intervention as reported in the papers.

## Results

The aim of this literature review was to establish, from the published corpus, how clinical leadership was developed among frontline healthcare providers. Interventions for clinical leadership were summarized and synthesized. A total of 24 papers exploring the implementation and the evaluation of interventions for clinical leadership development met the inclusion criteria. The interventions are summarized below. 

### Country where the intervention was implemented

All interventions for clinical leadership development included in this review were implemented in High-Income Countries (HIC). Thirteen papers reported on studies conducted in the United Kingdom (UK) (England, Ireland and Scotland) [25–37] while six reported studies in Australia [23, 38–42] and three in the United States of America (USA) [24, 43, 44]. One study was conducted in Belgium [45] and one in Switzerland [46] (Table 1).

**Table 1:** Country where the intervention was implemented

Author	Country	Year
Cleary et al. [38]	Australia	2005
Ferguson et al. [39]	Australia	2007
Williams et al. [40]	Australia	2009
Travaglia et al. [41]	Australia	2011
MacPhail et al. [23]	Australia	2015
Leggat et al. [42]	Australia	2016
Dierckx de Casterelé [45]	Belgium	2008
Miller and Dalton [25]	England	2011
Leeson and Millar [26]	England	2013
Enterkin et al. [27]	England	2013
Phillips and Byrne [31]	England	2013
Castillo and James [32]	England	2013
Stoll et al. [33]	England	2011
Miani et al. [34]	England	2013
Runnacle et al. [35]	England	2013
Lunn et al. [28]	Ireland	2008
McNamara et al. [29]	Ireland	2014
Fealy et al. [36]	Ireland	2015
Patton et al. [37]	Ireland	2013
Pearson et al. [30]	Scotland	2010
Martin et al. [46]	Switzerland	2012
Kling [43]	USA	2010
Abraham [44]	USA	2011
Lekan et al. [24]	USA	2011

### Aims of the interventions

The emphasis of most interventions was on developing clinical skills. Some interventions were designed to develop leadership competencies, to promote succession planning, to enhance the contribution of frontline healthcare providers to patient experiences, and to ensure quality and safe health services [23–31, 36, 37, 39–42, 44–46] (Table 2). Other interventions focused on preparing nursing students, medical students, and novice frontline healthcare providers for future leadership roles and for ensuring quality care and patient safety [33, 35, 43]. Some interventions were developed to transform managers into leaders [32].

**Table 2:** Aim of the intervention

Author	Country	Year	Aim of the intervention
Cleary et al. [38]	Australia	2005	Develop and consolidate clinical leadership skills
Ferguson et al. [39]	Australia	2007	Develop clinical leaders’ skills to observe clinical practices in a structured way to create a culture of quality and safety
Williams et al. [40]	Australia	2009	Develop necessary skills to act as clinical leaders
Travaglia et al. [41]	Australia	2011	Develop the skills to provide coordinated care
MacPhail et al. [23]	Australia	2015	Foster leadership capability and encourage engagement in decision making within their teams
Leggat et al. [42]	Australia	2016	Develop clinical leadership skills in ensuring high quality and safe health service
Dierckx de Casterelé [45]	Belgium	2008	Strengthen leadership competence in quality improvement projects
Miller and Dalton [25]	England	2011	Provide mentoring in clinical leadership
Leeson and Millar [26]	England	2013	Enable participants to take initiatives, focus on priorities and continuous quality improvement
Phillips and Byrne [31]	England	2013	Enhance ward managers’ contribution to patient experience and quality of care
Castillo and James [32]	England	2013	Transform managers into leaders
Stoll et al. [33]	England	2011	Develop future clinical leaders
Miani et al. [34]	England	2013	Foster a culture of quality improvement
Runnacle et al. [35]	England	2013	Prepare trainees to ensure safe and effective services
Enterkin et al. [27]	England	2013	Prepare participants for the role of ward sister
Lunn et al. [28]	Ireland	2008	Develop transformational leadership behaviours
McNamara et al. [29]	Ireland	2014	Develop clinical leadership skills
Fealy et al. [36]	Ireland	2015	Develop leadership competence to improve service delivery
Patton et al. [37]	Ireland	2013	Develop clinical leadership competencies
Pearson et al. [30]	Scotland	2010	Develop leadership potential
Martin et al. [46]	Switzerland	2012	Enhance leadership competence
Kling [43]	USA	2010	Maximize students learning
Abraham [44]	USA	2011	Enhance leadership skills
Lekan et al. [24]	USA	2011	Support the development of clinical leadership

### Target group for which interventions were implemented

Interventions for clinical leadership development targeted a variety of frontline health care providers (Table 3). Only a few interventions included frontline healthcare providers for maternal and child health [28–30, 32, 34, 36, 37, 41], while the remainder of interventions included early career nurses, qualified nurses, medical doctors, and allied healthcare professionals in hospital settings, including primary and secondary, acute, academic, community and regional hospitals, and mental health and geriatric wards [23, 26, 27, 31, 35, 38–40, 44, 45]. Other target groups include novice students, senior level nursing students, senior registrars, and postgraduate medical and dental students [24, 25, 42, 43].

**Table 3:** Target group which interventions were implemented

Author	Country	Year	Target group which interventions were implemented
Cleary et al. [38]	Australia	2005	Mental health nurses
Ferguson et al. [39]	Australia	2007	Clinical leaders in mental and occupational health, theatre, emergency, nursery, post-natal ward
Williams et al. [40]	Australia	2009	New graduates (nurses)
Travaglia et al. [41]	Australia	2011	Nursing and midwifery unit managers
MacPhail et al. [23]	Australia	2015	Medical doctors, nurses and allied health professionals
Leggat et al. [42]	Australia	2016	Medical doctors, nurses, and allied healthcare providers
Dierckx de Casterelé [45]	Belgium	2008	Head nurses
Miller and Dalton [25]	England	2011	Senior registrars
Leeson and Millar [26]	England	2013	Nurses and allied healthcare professionals
Enterkin et al. [27]	England	2013	Staff nurse and midwives, newly qualified nurses
Phillips and Byrne [31]	England	2013	Ward managers
Castillo and James [32]	England	2013	Wards managers, senior nurses and midwives
Stoll et al. [33]	England	2011	Junior doctors
Miani et al. [34]	England	2013	Doctors, nurses and midwives
Runnacle et al. [35]	England	2013	Trainees doctors
Lunn et al. [28]	Ireland	2008	Nurses and midwives
McNamara et al. [29]	Ireland		Nurses and midwives
Fealy et al. [36]	Ireland	2015	Nurses and midwives
Patton et al. [37]	Ireland	2013	Nurses and midwives
Pearson et al. [30]	Scotland	2010	Early career and qualified nurses and midwives
Martin et al. [46]	Switzerland	2012	Nurse leaders
Kling [43]	USA	2010	Novice students
Abraham [44]	USA	2011	Registered nurses
Lekan et al. [24]	USA	2011	Senior level nursing students

### Content areas covered by the interventions

Development of clinical skills was common to the majority of interventions as summarized in Table 4 [24, 28, 30, 33, 35–39, 41–43, 45, 46]. Other content areas included personal development, teamwork, team management, team building, service delivery, care processes, and the environment of care needed to ensure quality and safe services [23, 26, 27, 29, 31, 32, 34, 40, 44].

**Table 4:** Content areas covered by the interventions

Author	Country	Year	Content areas covered by the interventions
Cleary et al. [38]	Australia	2005	Personal development, teamwork, clinical skills, service delivery
Ferguson et al. [39]	Australia	2007	Observation, feedback skills, clinical practice skills, patient care, teamwork, environment of care, quality improvement (QI)
Williams et al. [40]	Australia	2009	Leadership skills development
Travaglia et al. [41]	Australia	2011	Clinical skills and leadership skills development
MacPhail et al. [23]	Australia	2015	Leadership skills, multi-disciplinary teamwork
Leggat et al. [42]	Australia	2016	Clinical skills leadership skills, quality and safety skills
Dierckx de Casterelé [45]	Belgium	2008	Clinical and leadership skills, teamwork, care environment, care giving process
Miller and Dalton [25]	England	2011	Teamwork
Leeson and Millar [26]	England	2013	Leadership skills development
Enterkin et al. [27]	England	2013	Leadership skills development
Phillips and Byrne [31]	England	2013	Clinical skills development, teamwork and patient care
Castillo and James [32]	England	2013	Leadership skills development, team management, and service improvement
Stoll et al. [33]	England	2011	Personal development, clinical skills, service delivery
Miani et al. [34]	England	2013	Clinical skills and leadership skills, team management and the environment of care
Runnacle et al. [35]	England	2013	Clinical skills
Lunn et al. [28]	Ireland	2008	Personal development, clinical skills, team building, patient care
McNamara et al. [29]	Ireland	2014	Clinical leadership skills development
Fealy et al. [36]	Ireland	2015	Clinical skills for service delivery
Patton et al. [37]	Ireland	2013	Personal development and teamwork
Pearson et al. [30]	Scotland	2010	Personal development, team management
Martin et al. [46]	Switzerland	2012	Clinical and leadership practice
Kling [43]	USA	2010	Clinical skills, personal development, leadership skills
Abraham [44]	USA	2011	Leadership skills development
Lekan et al. [24]	USA	2011	Leadership skills development, clinical skills, and patient care

### Educational approaches

Primarily the interventions for clinical leadership development were offered in the form of in-service training using a work-based learning (WBL) educational approach within the clinical settings [28, 29, 31–34, 36–38, 42, 45, 46]. Classroom-based learning (CBL) conducted in classrooms outside of clinical settings [30, 43] or a combination of both were also used [27, 41, 44]. Some interventions were offered as postgraduate training programmes, using a combination of WBL and CBL [24, 25, 30, 35] (Table 5).

**Table 5:** Educational approaches

Author	Country	Year	Educational approaches
Cleary et al. [38]	Australia	2005	Work-based learning (WBL) as in-service training
Ferguson et al. [39]	Australia	2007	WBL as in-service training
Williams et al. [40]	Australia	2009	WBL as in-service training
Travaglia et al. [41]	Australia	2011	Classroom based learning (CBL) as in-service training with online interaction sessions
MacPhail et al. [23]	Australia	2015	WBL as in-service training and demonstration of best practices
Leggat et al. [42]	Australia	2016	WBL and Case-Based Learning as in-service training
Dierckx de Casterelé [45]	Belgium	2008	WBL as in-service training
Miller and Dalton [25]	England	2011	WBL as postgraduate programme
Leeson and Millar [26]	England	2013	WBL as in-service training
Enterkin et al. [27]	England	2013	WBL and CBL as in-service training
Phillips and Byrne [31]	England	2013	WBL as in-service training
Castillo and James [32]	England	2013	WBL as in-service training
Stoll et al. [33]	England	2011	WBL as in-service training
Miani et al. [34]	England	2013	WBL
Runnacle et al. [35]	England	2013	WBL postgraduate training
Lunn et al. [28]	Ireland	2008	WBL as in-service training
McNamara et al. [29]	Ireland	2014	WBL as in-service training
Fealy et al. [36]	Ireland	2015	WBL as in-service training
Patton et al. [37]	Ireland	2013	WBL as in-service training
Pearson et al. [30]	Scotland	2010	CBL postgraduate programme
Martin et al. [46]	Switzerland	2012	WBL as in-service training
Kling [43]	USA	2010	CBL as in-service training
Abraham [44]	USA	2011	WBL and CBL as in-service training
Lekan et al. [24]	USA	2011	WBL and CBL Postgraduate programme

### Educational techniques

Interventions for clinical leadership development targeting frontline healthcare providers made use of a variety of educational techniques, used singularly or in combination (Table 6.) A combination of action learning, mentorship and coaching was used in six interventions to develop various skills [28–30, 33, 36, 37]. Other educational approaches included inquiry-based learning, self-directed learning, case-based learning, problem-based learning, experiential learning, and shadowing [23, 25–27, 29, 31, 32, 35, 38–40, 42, 44–46]. Clinical supervision was used only in one intervention [24].

**Table 6:** Educational techniques

Author	Country	Year	Educational techniques
Cleary et al. [38]	Australia	2005	Self-directed, Learning (SDL)
Ferguson et al. [39]	Australia	2007	Observation of clinical practice by clinical leaders, feedback and reflection
Williams et al. [40]	Australia	2009	Mentoring and role modelling (unit managers to new nurses)
Travaglia et al. [41]	Australia	2011	Coaching
MacPhail et al. [23]	Australia	2015	SDL, Problem-based learning (PBL)
Leggat et al. [42]	Australia	2016	Enquiry based learning (EBL), SDL
Dierckx de Casterelé [45]	Belgium	2008	Action learning
Miller and Dalton [25]	England	2011	Mentoring (senior managers to registrars)
Leeson and Millar [26]	England	2013	PBL
Enterkin et al. [27]	England	2013	SDL, Action learning
Phillips and Byrne [31]	England	2013	CBL, Action learning
Castillo and James [32]	England	2013	Coaching Action learning
Stoll et al. [33]	England	2011	Coaching, Mentoring Action learning, QI projects
Miani et al. [34]	England	2013	Experiential learning, QI projects
Runnacle et al. [35]	England	2013	Experiential learning, QI projects
Lunn et al. [28]	Ireland	2008	Experiential learning, Action learning, Coaching, Shadowing
McNamara et al. [29]	Ireland	2014	Action learning Mentoring Coaching
Fealy et al. [36]	Ireland	2015	SDL Mentoring Coaching Action learning
Patton et al. [37]	Ireland	2013	SDL, Action Learning, Mentoring, Coaching
Pearson et al. [30]	Scotland	2010	Coaching, Mentoring, Action learning sets
Martin et al. [46]	Switzerland	2012	Case Based Learning (CBL) Coaching Action learning
Kling [43]	USA	2010	Peer mentoring (senior students to novice students)
Abraham [44]	USA	2011	Experiential learning
Lekan et al. [24]	USA	2011	Bedside clinical teaching. Clinical supervision

### Time frame of interventions for clinical leadership development

Most interventions for clinical leadership development were offered as multiple contact sessions of varying duration, ranging from a few days, to a few weeks, or to lasting several months [24, 26, 30, 33, 37–40, 44–46] (Table 7). Other interventions were offered as multiple contact sessions in postgraduates programmes [24, 30, 35]. One intervention was offered as a full-time master degree programme with no detail of the contact sessions provided [25].

**Table 7:** Time frame of interventions for clinical leadership development

Author	Country	Year	Time frame of interventions for clinical leadership development
Cleary et al. [38]	Australia	2005	6 months (nature and length of contact sessions missing)
Ferguson et al. [39]	Australia	2007	12 observations over 4 months
Williams et al. [40]	Australia	2009	4 full time intensive weeks
Travaglia et al. [41]	Australia	2011	5 face-to-face days and monthly collaborative coaching over 24 months (length of coaching sessions missing)
MacPhail et al. [23]	Australia	2015	One 2-h session once per month for 9 months
Leggat et al. [42]	Australia	2016	12 months (length of contact sessions
Dierckx de Casterelé [45]	Belgium	2008	12 months (nature and length of contact sessions missing)
Miller and Dalton [25]	England	2011	1-year full time master’s programme (nature and length of contact sessions missing)
Leeson and Millar [26]	England	2013	2 days per week, every week over 6 weeks
Enterkin et al. [27]	England	2013	8 days, 1 day/ month over 8 months
Phillips and Byrne [31]	England	2013	Four modules, each 8-h days per day
Castillo and James [32]	England	2013	Three 1-day module, three ½ day action learning sets over 8 months
Stoll et al. [33]	England	2011	12 months full time programme (nature and length of contact sessions missing)
Miani et al. [34]	England	2013	2 days (internal fellows), and 4 days a week external fellows) over 12 months
Runnacle et al. [35]	England	2013	1-h workshop; 6 months programme (2 full workshops 1 month apart; over 1-year full time fellowship
Lunn et al. [28]	Ireland	2008	12 months (nature and length of contact sessions missing)
McNamara et al. [29]	Ireland	2014	6 months’ (nature and length of contact sessions missing)
Fealy et al. [36]	Ireland	2015	6 months tailored to participants needs
Patton et al. [37]	Ireland	2013	Tailored to individuals’ time frame over 6 months
Pearson et al. [30]	Scotland	2010	1 year flying start, 2 years masters’ degree periodically, Action learning sets, over three years
Martin et al. [46]	Switzerland	2012	18 days (1 day/month) over 12 months for the intervention phase and over 6 months in the follow up phase
Kling [43]	USA	2010	3 h’ classroom, 6 h per week clinical component
Abraham [44]	USA	2011	32 h/month over 6 months
Lekan et al. [24]	USA	2011	3 weeks SDL and CBL, 3 weeks 8- h clinical rotation. 1-week reflective journal

### How interventions were measured

Best practice in measuring an intervention is to use pre-post evaluation. Nine out of twenty-four studies used pre-and post-test methods to measure the learning attainment, behaviour, and impact of the intervention [23, 24, 28, 35, 38, 41, 42, 44, 46]. Fifteen studies used only post-test methods to measure the effectiveness of the interventions (Table 8).

**Table 8:** How interventions were assessed

Author	Country	Year	How interventions were assessed
Cleary et al. [38]	Australia	2005	Pre-and post-assessment to measure the reaction, and learning attainment of participants using the Nurse Self-Concept Questionnaire
Ferguson et al. [39]	Australia	2007	Post-test to measure the learning attainment and behaviour of participants using review of observation documents
Williams et al. [40]	Australia	2009	Post-test to measure the reaction participants using a Questionnaire and focus group discussions (FDGs)
Travaglia et al. [41]	Australia	2011	Mid-term assessment to measure the learning attainment and behavior of participants, and impact on service delivery interviews and online survey
MacPhail et al. [23]	Australia	2015	Pre-and post-assessment and follow-up 18 moths post intervention to assess the reaction, and learning attainment of participants using structured evaluation survey and questionnaire
Leggat et al. [42]	Australia	2016	Pre-and post-assessment to measure participants behaviour and impact of the intervention using questionnaires and interviews
Dierckx de Casterelé [45]	Belgium	2008	Post-test assessment to measure the behaviour, learning attainment and impact of the intervention, using interviews, FDGs and observation of participants
Miller and Dalton [25]	England	2011	Post-test assessment to measure individuals’ reaction using FDGs, interviews, and online questionnaires
Leeson and Millar [26]	England	2013	Post-test assessment to measure the reaction and learning attainment, and behaviour of participants, using evaluation sheets
Enterkin et al. [27]	England	2013	Post-test assessment to measure participants’ reaction and learning attainment questionnaires
Phillips and Byrne [31]	England	2013	Post-test assessment to measure the reaction and learning of participants, using questionnaires
Castillo and James [32]	England	2013	Post-test assessment to measure participant reaction, learning, behaviour and impact of the intervention using questionnaires
Stoll et al. [33]	England	2011	Post-test assessment to measure, learning attainment and impact of the intervention, using questionnaires and interviews
Miani et al. [34]	England	2013	Post-test assessment to measure the learning of participant, behaviour, and impact of the intervention, using Online questionnaires and interviews
Runnacle et al. [35]	England	2013	Pre- and post- assessment to measure the reaction of participants
Lunn et al. [28]	Ireland	2008	Pre-and post-assessment to measure the reaction, learning and behaviour of participants, using questionnaires
McNamara et al. [29]	Ireland	2014	Post-test assessment to measure participants’ reactions using FDGs, and interviews
Fealy et al. [36]	Ireland	2015	Post-test assessment to measure the impact of the intervention, using service assessment tools
Patton et al. [37]	Ireland	2013	Post-test assessment to measure participants’ learning and behaviour using the leadership practice inventory, clinical leaders’ behaviour questionnaires, FGDs and group interviews
Pearson et al. [30]	Scotland	2010	Post-test assessment to measure participants’ reaction and behaviour, using FGDs and questionnaires
Martin et al. [46]	Switzerland	2012	Pre-and post- assessment to participants’ behaviour using, observation and self-assessment tools
Kling [43]	USA	2010	Post-test assessment at 6-month post intervention to measure participant reaction, learning attainment and behaviour using questionnaires
Abraham [44]	USA	2011	Pre-and post- assessment at 6 and 12 months following completion of intervention to measure participant learning, behaviour and impact of the intervention
Lekan et al. [24]	USA	2011	Pre-and post- test assessment to measure participant reaction, learning attainment, and impact of the intervention

To categorize how the different articles evaluated their interventions, Kirkpatrick’s approach was used. Only one study included an evaluation at all four levels namely, the reaction, learning attainment and behaviour, and impact of the intervention on service delivery [32]. Measuring participant reactions to the interventions was common to most interventions [23–27, 29–32, 35, 38–40, 43]. Learning attainment, and the behavior of participants were also measured. The tools used to collect evaluation data included self-report questionnaires, online surveys, evaluation sheets, structured evaluation forms. Additional tools included in-depth-interviews, group interviews, FGDs, observations of action learning sets and document review.

### Outcomes of the interventions as reported in the papers

The outcomes of the interventions recorded in the papers include: personal development [increased self-awareness and confidence, feelings of empowerment, time management, development of emotional intelligence skills and increased learning ability] [27, 32, 34, 37, 38, 45]; enhanced leadership knowledge and skills [communication, willingness to lead teams, delegation, ability to empower others, problem solving, decision making, ability to inspire a shared vision, team management] [24, 26–29, 32, 34–37, 41, 43–46]; improved clinical knowledge and skills [enhanced basic nursing knowledge and skills, improved clinical practices, understanding of contribution to patient care] [42], improved teamwork [ability to work as part of multi-disciplinary teams, ability to manage teams] [23, 25, 30, 31, 37, 40, 43], improved patient care [increased focus on patient care, improved patient outcomes], and service delivery [change in care processes] [24, 28, 33, 39, 41, 45] (Table 9).

**Table 9:** Outcomes of the interventions as reported in the papers

Author	Country	Year	Outcomes of the interventions as reported in the papers
Cleary et al. [38]	Australia	2005	Intervention useful to their work, Improved communication, clinical skills, teamwork
Ferguson et al. [39]	Australia	2007	Opportunity to review care practices, development of QI plans, improved observation and feedback skills, team building
Williams et al. [40]	Australia	2009	Evaluated positively bay all stakeholders
Travaglia et al. [41]	Australia	2011	Feeling of empowerment to implement change in the work environment, improved communication, unit performance and patient flow
MacPhail et al. [23]	Australia	2015	High satisfaction with the intervention, feasible, increased willingness to lead teams and work as part of multidisciplinary teams
Leggat et al. [42]	Australia	2016	Improved leadership practices, emotional intelligence, psychological empowerment patient safety skills
Dierckx de Casterelé [45]	Belgium	2008	Self-awareness enhanced communication skills, improvement of the work environment
Miller and Dalton [25]	England	2011	Successful in building teamwork and communication
Leeson and Millar [26]	England	2013	Positive experience, ability to take responsibility for action, change in working practices
Enterkin et al. [27]	England	2013	Feelings of empowerment, self-awareness and confidence, ability to delegate, and empower others, feeling of support from management
Phillips and Byrne [31]	England	2013	Increased understanding of participants’ contribution to patient care
Castillo and James [32]	England	2013	Improved confidence, better communication, increased problem-solving skills
Stoll et al. [33]	England	2011	Greater understand of service delivery, change in care processes and procedures
Miani et al. [34]	England	2013	Enhanced leadership and communication skills, team management skills, increased confidence, improved patient experience
Runnacle et al. [35]	England	2013	Improvement in use of quality improvement skills
Lunn et al. [28]	Ireland	2008	Enhanced communication, problem solving and decision-making skills, ability to empower teams
McNamara et al. [29]	Ireland	2014	Supportive and contributes to clinical leadership skills development
Fealy et al. [36]	Ireland	2015	Service development, improved care practices
Patton et al. [37]	Ireland	2013	Increased self-awareness, improved communication skills and team work
Pearson et al. [30]	Scotland	2010	Good in preparing participants for work challenges, increased ability to manage relationships
Martin et al. [46]	Switzerland	2012	Improved ability to inspire shared vision, and challenging the process
Kling [43]	USA	2010	Positive experience, enhanced basic nursing skills and knowledge, improved time management and delegation skills
Abraham [44]	USA	2011	Improved decision-making skills
Lekan et al. [24]	USA	2011	Improved communication skills, ability to delegate, skills to lead practice, patient outcomes, promotion of nurses to supervisory posts

### Limitations of the interventions

Of studies that reported the limitations of interventions the following were identified: difficulty in gaining consent from patients to be observed while care was being provided and some trainers may not be skilled enough to observe using direct observation [39]; interventions that were too intensive and demanding, affecting the motivation and ability of participants to attend all sessions [27, 30, 40]; time away from clinical duties, resistance from colleagues to implement changed practices, and nurses or midwives taking clinical leadership roles and lack of support from health service managers [23, 25, 34, 41]; short timeline for progamme implementation which did not allow for assessing the impact of interventions on participants, service users and on service delivery [23, 29, 34, 36]; and challenges with sustainability of gains made through the interventions [31, 38]. A lack of a control group in evaluating interventions was also considered a limitation in attributing changes to the intervention [24]. The transferability of the intervention was also questioned [42] (Table 10).

**Table 10:** Limitations of the interventions

Author	Country	Year	Limitations of the interventions
Cleary et al. [38]	Australia	2005	Sustainability of the intervention is challenging
Ferguson et al. [39]	Australia	2007	Difficulty in gaining consent from patient to be observed; skills of observer
Williams et al. [40]	Australia	2009	Too intensive thus affecting motivation and ability to attend all sessions
Travaglia et al. [41]	Australia	2011	Resistance from colleagues to change, and nurse/ midwives taking clinical leadership roles, time constraints
MacPhail et al. [23]	Australia	2015	Time away from clinical duties Short timeline from progamme implementation and limited evaluation of participants’ leadership knowledge and skills
Leggat et al. [42]	Australia	2016	*
Dierckx de Casterelé[45]	Belgium	2008	*
Miller and Dalton [25]	England	2011	Time away from the clinical setting
Leeson and Millar [26]	England	2013	*
Enterkin et al. [27]	England	2013	Intervention too long
Phillips and Byrne [31]	England	2013	Maintaining momentum generated by the intervention
Castillo and James [32]	England	2013	*
Stoll et al. [33]	England	2011	*
Miani et al. [34]	England	2013	Resistance to change from frontline healthcare providers who did not taking part in the programme. Short period of time to enable change
Runnacle et al. [35]	England	2013	*
Lunn et al. [28]	Ireland	2008	*
McNamara et al. [29]	Ireland	2014	*
Fealy et al. [36]	Ireland	2015	*
Patton et al. [37]	Ireland	2013	*
Pearson et al. [30]	Scotland	2010	Intervention very demanding
Martin et al. [46]	Switzerland	2012	
Kling [43]	USA	2010	*
Abraham [44]	USA	2011	*
Lekan et al. [24]	USA	2011	Without control group changes cannot be conclusively attributed to the intervention

*represents missing data

## Discussion

This literature review of the implementation and evaluation of interventions for clinical leadership development was conducted towards identifying a model to inform clinical leadership development among frontline healthcare providers in Low- and Middle-Income Countries (LMICs) generally, and for the delivery of optimal maternal and perinatal care in South Africa specifically.

All descriptions of interventions for clinical leadership development derive from studies implemented in HICs. This would limit the transferability of study findings to LMICs, where clinical leadership is still underdeveloped and healthcare systems are faced with different contextual challenges [8]. Studies are required to explore appropriate interventions to improve clinical leadership in LMICs, including South Africa.

Of note, clinical leadership development programmes targeted novice to veteran frontline healthcare providers, in both formal and informal leadership positions [23]. This could indicate a previous neglect of ongoing clinical leadership development amongst frontline healthcare workers across the health system. With the emphasis on developing clinical expertise, interventions for clinical leadership development must include frontline healthcare workers who have been practicing for some time and may serve the purpose of updating veteran healthcare workers to new evidence-based practices of care.

Some interventions for clinical leadership development reported in this review embraced a holistic conceptualization of clinical leadership, paying attention to clinical skills, leadership skills, team building, team management, the environment of care, and service delivery [34, 38, 45]. Other interventions were more selective, based on checklists of whether participants manifested certain clinical skills. Interventions that embrace a holistic conceptualization of clinical leadership are more detailed, and can produce well trained and skilled clinical leaders. However, they may be expensive, and may require longer training periods, as they include multiple dimensions of clinical leadership. Interventions based on a selective understanding of clinical leadership may be shorter in nature, as they may focus on fewer dimensions of clinical leadership. However, these interventions may not be able to produce skilled clinical leaders.

Most interventions for clinical leadership development used work-based learning as an educational approach to improve, develop, maintain or increase practicing professionals’ competence in the clinical setting [47, 48]. Work-based learning (WBL) has been shown to promote practical learning and to help practitioners relate new knowledge to their work environment [49, 50]. Classroom-based learning takes participants away from their work environment, a feature often considered as a major weakness of this approach [49, 50]. A systematic review evaluating in-service training suggests that WBL is the most appropriate approach to improve not only the knowledge of participants but also the skills, behaviors and attitudes of participants [51–53]. WBL with experiential teaching techniques, such as mentoring and coaching, can ensure effective clinical leadership development of frontline healthcare providers.

In many interventions, the actual length of exposure to contact sessions, and the balance of time between the delivery of training content, and hands-on activities, were not detailed. The paucity of information poses a challenge when trying to replicate the interventions to other settings. In the interventions that did indeed describe the length of exposure to the intervention, multiple contact sessions, over varying periods of time, were used to deliver the interventions. Intensive once-off training sessions are shown to have a negative impact on participants’ motivation [27, 40]. Multiple time-spaced contact sessions appear to be the most suitable approach to delivering in-service training programmes, as they provide participants with sufficient time and space to engage, reflect on the content of the training programme, and apply knowledge and skills to the work place [52, 54]. While designing interventions for clinical leadership development, there is a need to ensure that a reasonable timeframe tailored to participants’ needs is provided.

Most studies used only post-test evaluation to measure the effectiveness of the interventions. Post-test evaluation is outcome oriented and is concerned with the results of the intervention. The absence of pre-test observations and a lack of a control group in post-test evaluations limits the ability to attribute observed changes to the intervention [55]. Nonetheless, post-test is used in most interventions because of the logistical difficulties in obtaining pre-test observations due to time constraints [56].

Pre-post-test evaluation may be the most accurate way to provide a full picture of changes in participants over the course of the training programme. [56]. However, many interventions were implemented as once-off short interventions, over a couple of weeks. A short implementation timeline may not be sufficient to allow change to occur, and may not permit sufficient time to measure the impact of interventions in participants, teams, environments of care, or service delivery [23, 30, 34, 36].

Kirkpatrick’s approach to evaluation recommends four levels of evaluation to objectively measure the effectiveness of training programmes [19]. Most papers did not provide thorough descriptions of evaluation methods. Only one study reviewed included an evaluation at each of the four levels suggested by Kirkpatrick’s approach [32]. Most papers reporting the evaluation of interventions for clinical leadership development focused on the reactions of participants and learning attainment [27, 32, 34, 37, 38, 45]. Participants reported positive experiences, and indicated the acquisition of leadership knowledge and skills as result of the intervention [27, 32, 34, 37, 38, 45]. Some studies reported improved clinical knowledge and skills improved teamwork as the behavior of participants [23, 25, 29, 33, 34, 40, 42, 43]. The impact of interventions include improved patient care, improved patient outcomes, and change in care processes [24, 28, 33, 39, 41, 45].

Although some interventions used validated tools to evaluate the interventions, most outcomes recorded in this review used self-reported changes. Tools that elicit self-reported learning attainment and behaviour changes are considered to provide weak evaluation evidence and are of variable accuracy [57]. Factors that affect accuracy include information bias, influenced by recall bias and social desirability bias, and design bias, influenced by questionnaire design and mode of data collection [57]. To move beyond the weaknesses of to self-reported changes, the literature suggests the use of 360° assessments [58–60]. This method involves an individual and several other people (e.g. peers, supervisors, assessors, and managers) provide a comprehensive feedback on an individual’s behaviour and effectiveness [60]. It is suggested that used in combination with training programmes or interventions, 360° feedback can be an effective assessment tool [58–60]. Adequate descriptions of interventions, and rigorous description of methods used in implementing, and evaluating the interventions are required to ensure transferability of findings of interventions to other settings.

Most studies did not discuss the limitations of the interventions, or the sustainability of gains made through the intervention. One strategy to ensure sustainability of interventions for clinical leadership development is the team training approach [23]. A team training approach to clinical leadership development may serve a dual purpose: the transfer of skills and teambuilding. Teambuilding is an integral part of clinical leadership development, as well as an outcome of clinical leadership. A team training approach allows multiple professionals to be trained together, reduces resistance to change, and reduces the resistance to frontline healthcare leaders taking clinical leadership roles [23].

### Strengths of the review

This review highlights the diversity, extent, and gaps of interventions for the development of clinical leadership among frontline healthcare providers. The review also highlights the conceptualizations of clinical leadership embedded in the interventions, and the challenges encountered in the implementation of interventions for clinical leadership development.

### Limitations of the review

Although rigorous steps were carried out in this review, we are also aware of some limitations

- Studies may have been omitted from the review if they were not published in the databases searched, or if they were published in languages other than English.
- The choice to limit the search to articles that described the implementation or evaluation of interventions for clinical leadership development among frontline healthcare providers, and published between 2004 and 2017, may have reduced the range of articles included in the review.

## Conclusions

The literature review was conducted towards identifying a model to inform clinical leadership development among frontline healthcare providers in LMIC settings. All studies reviewed arose in HIC settings, demonstrating the need for studies on frontline clinical leadership development in LMIC settings. The synthesis of studies conducted in HIC setting revealed what needs to be considered in the design of clinical leadership development interventions in LMIC settings. Firstly, clinical leadership development is an on-going process and must target both novice and veteran frontline health care providers. Secondly, the content of clinical leadership development interventions must encompass a holistic conceptualization of clinical leadership, with a focus on clinical skills and on competencies that support optimal clinical care. Thirdly, interventions for clinical leadership development should use work-based learning approaches, and experiential and practice-based learning techniques, as these are more likely to contribute to the sustainable development of clinical leadership among frontline healthcare providers, and to the improvement in overall service delivery. Fourthly, team-based approaches to clinical leadership development, implemented through multiple contacts over a period of time, allow the acquisition and the transfer of skills, and teambuilding. Fifthly, assessment of the expected learning and evaluation of expected outcomes need to be carefully planned in the design of clinical leadership development interventions, and measured preferable through pre-post assessments, and 360^0^ assessments. Lastly, adequate description of the implementation setting, of the intervention model, and of the methods used in implementing and evaluating the interventions are necessary to ensure transferability of an intervention to other settings. These guidelines established from this review of the literature, must be incorporated in the design of interventions for clinical leadership development in LMIC settings.

## Abbreviations

HICs: High-Income Countries
LMICs: Low-and-Middle-Income Countries
StaRI: Standards for Reporting Implementation Studies
UK: United Kingdom
USA: United States of America
WBL: Work based learning
CBL: Classroom based learning
SDL: self-directed learning
FGDs: focus group discussions
